# Open-Book Versus Closed-Book Tests in University Classes: A Field Experiment

**DOI:** 10.3389/fpsyg.2019.00463

**Published:** 2019-03-15

**Authors:** Ralf Rummer, Judith Schweppe, Annett Schwede

**Affiliations:** ^1^ Department of Psychology, University of Kassel, Kassel, Germany; ^2^ Department of Psychology, University of Erfurt, Erfurt, Germany; ^3^ Bundesarbeitsgericht, Erfurt, Germany

**Keywords:** testing effect, retrieval practice, open-book test, closed-book test, classroom study

## Abstract

The present field study compared open-book testing and closed-book testing in two (parallel) introductory university courses in cognitive psychology. The critical manipulation concerned seven lessons. In these lessons, all students received two to three questions concerning the content of the respective lesson. Half the participants (open-book group) were allowed to use their notes and the course materials, which had been distributed at the beginning of each class; the other half was not allowed to use these materials (closed-book group). A surprise test conducted in the eighth week demonstrated better results for the closed-book group. Further 6 weeks later, the final module exam took place. A number of questions in this exam concerned the learning matters instructed during the critical seven lessons. Even with respect to these questions, the closed-book group performed better than the open-book group. We discuss these results with respect to two possible explanations, retrieval practice and motivational differences.

## Introduction

The *testing effect* is one of the most prominent instances of difficulties during learning, being desirable for long-term learning (Bjork, 1994; Roediger and Karpicke, 2006). More than a century ago, first studies have demonstrated that active retrieval of previously studied information is a more useful strategy to prevent forgetting than passive restudying (e.g., Witasek, 1907; Abott, 1909; Kühn, 1914; Gates, 1917). During the last years, this finding, which also has been termed *retrieval practice effect* or *quizzing effect*, received growing attention and became one of the most investigated empirical phenomena in (applied) cognitive psychology (for comprehensive overviews cf. Rowland, 2014; Adesope et al., 2017). Effects of retrieval practice were demonstrated with different learning materials (e.g., vocabularies, texts, multimedia materials) as well as in different contexts (laboratory, school classes, university classes, etc.; see Dunlosky et al., 2013).

Many (laboratory) studies concerned with the testing effect follow a typical procedure: First, participants are presented with the learning material, regularly a brief science text. This text has to be learned under two different conditions: In the control condition, participants are instructed to read the text several times; in the critical testing condition, however, they have to read the text once and then recall its content (and sometimes for several times, e.g., Roediger and Karpicke, 2006, Exp. 2). In some of the experiments, participants do not have the opportunity to reread the text after the testing phase and no feedback is provided (e.g., Roediger and Karpicke, 2006; Rummer et al., 2017, Exp. 1); in other studies, the opportunity to reread the text after the testing phase provides indirect feedback (e.g., McDaniel et al., 2009; Karpicke and Blunt, 2011; Rummer et al., 2017, Exp. 2). The learning phase is followed by the final test, often contrasting an immediate test (usually after 5 min) with a delayed one (usually after 1 week). The findings of these experiments sometimes demonstrate no testing effect or even a moderate learning advantage for rereading over testing in the immediate final test condition (5 min after the learning phase) but a strong and robust advantage for the testing condition for those participants tested after 1 week (e.g., Wheeler et al., 2003; Roediger and Karpicke, 2006; Toppino and Cohen, 2009; Bouwmeester and Verkoeijen, 2011; Congleton and Rajaram, 2012; Rummer et al., 2017). Rowland’s (2014) meta-analysis on laboratory studies following this procedure revealed a medium-sized testing effect with larger effect sizes for retention intervals of at least 1 day compared to retention intervals less than 1 day and when feedback was provided compared to conditions without feedback.

The testing effect was not solely investigated in lab studies but also in schools (e.g., Carpenter et al., 2009; McDaniel et al., 2011; Roediger et al., 2011) and universities (e.g., Lyle and Crawford, 2011; Batsell et al., 2017). For instance, Batsell et al. (2017) explored the external validity of the testing effect in two introductory psychology courses. They compared reading assignments and reading assignments plus quizzes on textbook chapters that were otherwise not covered in class. Performance on three exams during the term served as the dependent variable. The quiz group scored significantly higher than the control group on the same questions, on similar questions, and even on new questions. Lyle and Crawford (2011) compared two sections of an undergraduate course on statistics for psychology in consecutive years taught by the same instructor. In the second year, students took the final 5–10 min of each class to answer 2–6 short-answer questions addressing the same day’s lecture without consulting their notes. Performance on these quizzes accounted for 8% of students’ overall course grade. The scores on four non-cumulative exams were higher in the section with practice tests than in the one without.

Overall, these studies indicate that the testing effect translates to naturalistic learning settings. This conclusion is further supported by a meta-analysis on the testing effect in psychology classrooms (Schwieren et al., 2017), which also revealed a medium-sized effect. The presence of feedback also moderated the testing effect substantially in these studies; retention interval was not included as a moderator variable.

One of the most widespread explanations for the beneficial effect of testing is in terms of retrieval practice: active retrieval from memory is assumed to foster deeper processing more effectively than repeated passive learning and to strengthen the memory trace by elaborating the encoded information and by creating different retrieval routes to the information in long-term memory (e.g., Carpenter and DeLosh, 2006; Carpenter, 2009). The testing effect can also be explained in terms of transfer-appropriate processing (Morris et al., 1977): From this perspective, practicing retrieval during learning is beneficial because it is more similar to the processing required at test than is restudying (e.g., Bjork, 1988; Roediger and Karpicke, 2006).

Testing is also assumed to have indirect effects on future learning behavior because it provides diagnostic information on the current state of learning. Learners can use this information to adapt subsequent learning activities, for instance, by investing more time and/or effort because of failed retrieval or by applying more elaborative encoding strategies (Fernandez and Jamet, 2017). It is plausible to assume that these indirect effects are particularly important in naturalistic learning settings in which the outcomes of learning are relevant, for instance in an exam. Most researchers assume that the testing effect is due to more than one mechanism and that the role of these mechanisms depends on the characteristics of the specific testing procedure as well as on the learning materials and setting (Rowland, 2014).

A potential methodological issue is that most studies compare testing only to a passive restudy control condition, as in the typical procedure described above (Kornell et al., 2012). This is particularly the case for field experiments, in which often even a no restudy control condition is used (cf. Schwieren et al., 2017). Given that merely rereading a text is not very effective in itself, this may result in an overestimation of the size and the importance of the testing effect (Kornell et al., 2012). However, there are a few laboratory studies that demonstrate the effectiveness of testing even over more elaborative control conditions such as concept mapping (Karpicke and Blunt, 2011; Blunt and Karpicke, 2014; for a replication of this finding cf. also Camerer et al., 2018) or note-taking (McDaniel et al., 2009; Dobson and Linderholm, 2015; Nguyen and McDaniel, 2016; Rummer et al., 2017). Remarkably, though, these beneficial effects did not always show up at the typical final test delay of 1 week, but only after a 2 weeks’ delay (Rummer et al., 2017, Exp. 1).

One active control condition that is particularly informative when it comes to retrieval practice as the source of the testing effect is open-book testing (Agarwal et al., 2008; Agarwal and Roediger, 2011; Gharib et al., 2012; Roelle and Berthold, 2017). Here, students are allowed to consult their notes or textbooks while taking the (practice) test. With respect to retrieval from long-term memory, open-book tests thus represent a very strict control condition in that the closed-book group and the open-book group practice answering the same questions but only the closed-book group is required to come up with the answers from memory. Though retrieval practice is not excluded with an open-book test, it should be much less likely than with a closed-book test. Surprisingly, however, previous studies comparing open-book and closed-book tests (with feedback), have not found any differences. Agarwal et al. (2008) had their participants study prose passages in six (Exp. 1) or eight (Exp. 2) different conditions in a within-subject design, four of which included practice tests: study plus closed-book test, study plus closed-book test with feedback, study plus open-book test, and study and take an open-book test simultaneously. The final test, which took place 1 week later, did not reveal any systematic differences between open-book tests and closed-book tests with feedback. Agarwal and Roediger (2011) replicated these findings for comprehension questions (identical with the practice questions) and transfer questions that required participants to indicate why a certain detail from the passage was true (after a 2-day final test delay).

Roelle and Berthold (2017) also applied a closed-book test versus open-book test manipulation while additionally varying the complexity of the questions. Their participants read expository texts and then either answered summarization or inference questions, either with or without the opportunity to reinspect the text. In addition, they varied the delay of the final test (immediate vs. 1 week later), which included the practiced summarization and inference questions as well as transfer questions. They observed an advantage of closed-book tests for practicing with summarization questions, but an advantage of open-book tests for practicing with inference questions. It is, however, noteworthy that the pattern looked slightly different when only the critical delayed final test is considered (Roelle, personal communication). Here, participants who practiced with summarization questions showed a closed-book advantage for all question types in the final test, while the open-book advantage for practicing with inference questions was restricted to the inference questions and the transfer questions.

There is also one field experiment investigating the benefits of open-book versus closed-book tests, which was conducted with students in an introductory psychology course (Gharib et al., 2012). Here, the manipulation was slightly different in that actual exam types (closed-book, open-book, and cheat sheet) were manipulated rather than no-stakes or low-stakes practice tests. For the question of a retrieval practice effect, students’ performance in a surprise retention quiz 2 weeks after the exams constituted the critical dependent variable. In line with the previously mentioned studies, the exam type did not influence performance in the surprise retention quiz.

In sum, these studies on open-book versus closed-book testing do not support the assumption that the explicit retrieval demand in closed-book tests results in better performance compared to open-book tests, which can be taken without retrieving information from memory. From a retrieval practice perspective, the lack of such a closed-book advantage effect is surprising. One reason for the lack of a consistent effect might be the combination of an elaborative control condition and a (rather) short retention interval. As indicated above, Rummer et al. (2017), Exp. 1, found an advantage of testing over note-taking only when the final test was delayed by 2 weeks, whereas participants who took notes and who recalled text information performed equally well on a final test after 1 week (and note-taking outperformed testing in an immediate test). Note-taking and open-book tests are similar in that learners process the material more elaborately and more actively than during rereading but do not need to retrieve any information from memory since the material is at hand. Thus, final test delays of up to 1 week might have been too short to detect a retrieval practice effect compared to open-book testing. The study by Gharib et al. (2012) used a delay of 2 weeks but their findings are not easily comparable to the other studies, as the manipulation did not concern a learning condition in terms of a practice test but an exam condition: students had either access to their notes, to a self-prepared cheat sheet, or to none of the former in the exam. In this case, potential differences due to the actual exam might have been obscured because students prepare differently for closed-book and for open-book exams (e.g., Theophilides and Koutselini, 2000).

Furthermore, Agarwal et al. (2008), p. 872, speculate that “any positive effects of closed-book tests may be even more powerful in a repeated testing design” such that repeated closed-book tests with feedback may result in better long-term retention than repeated open-book tests. To our knowledge, all previous studies contrasting open-book and closed-book tests have applied only a single testing phase with a single text.

Our study aims at further investigating potentially different effects of open-book and closed-book tests but under conditions that should maximize the impact of closed-book tests, that is, with a rather long final test delay and several practice tests (though not repeated for the same text). Furthermore, we address this question in a naturalistic learning setting in two introductory cognitive psychology courses and over a learning phase spanning 7 weeks. Under these conditions, we expect a retrieval practice effect, that is, better final test performance for the closed-book group than for the open-book group.

## Experiment

This field experiment was based on a one-factorial between-subjects design with the independent factor practice test type (open-book vs. closed-book). The experiment took place during 8 weeks of two parallel introductory seminars in cognitive psychology at the University of Erfurt (title: Cognition) taught by the third author, with one course serving as the experimental group (closed-book tests) and the other as the control group (open-book tests). Seven lessons of each course ended with a practice test on the current lesson. The experimental group and the control group received the same practice tests, but students in the control group could consult provided documents while answering the questions (open-book tests) and students in the experimental group could not (closed-book tests). The main dependent variable was performance on a surprise quiz in week 8. Performance in a final module exam, which took place 8 weeks after the surprise quiz and included a number of questions addressing the learning matter taught in the seven critical seminar lessons, served as an additional dependent variable. The experimental procedure is summarized in Table 1.

**Table 1:** Timeline of the two seminars.

Lesson(s)	Content of the respective lesson
Introductory Lessons	Introduction to the seminar, introduction to reading of scientific papers, signing consents, etc.
**Lesson 1**	**First experimental session (including one presentation on the “architecture of working memory”;** Baddeley, 2000**)**
**Lesson 2**	**Second experimental session including two presentations on “memory for sentences” (**Jarvella, 1971; Potter and Lombardi, 1990**)**
**Lesson 3**	**Third experimental session including two presentations on the “DRM paradigm” (**Roediger and McDermott, 1995; Meyersburg et al., 2009**)**
**Lesson 4**	**Fourth experimental session including one presentation on modality and DRM memory (**Kellogg, 2001**) and one on false memories in eye-witness reports (**Loftus et al., 1978**)**
**Lesson 5**	**Fifth experimental session (including one presentation on verbal overshadowing,** Melcher and Schooler, 1996**, and one on the Cognitive Load Theory,** Sweller, 1994**)**
**Lesson 6**	**Sixth experimental session (including two presentations on the “Cognitive Theory of Multimedia Learning” (**Mayer and Moreno, 1998; Moreno and Mayer, 1999**)**
**Lesson 7**	**Seventh experimental session including one presentation on the attentional and perceptual basis of the modality effect (**Rummer et al., 2011**).**
*Lesson 8*	*Surprise quiz including the same questions presented at the end of the lessons 1–7*
Lessons 9 to 12	Regular lessons without any experimental manipulation
*Additional meeting 8 weeks after the surprise quiz*	*Final Module Exam (60 min) including 9 multiple choice questions related to the lessons 1–7 but not asked before and 21 multiple choice questions unrelated to lessons 1–7.*

The lessons in which the practice tests (open-book tests versus closed-book test) were presented are highlighted using bold font; those lessons in which the two dependent measures took place (surprise quiz and final module exam) are set in italics.

## Method

### Participants

All the participants were undergraduate students of psychology in their first semester. They were informed that the two seminars were part of a research project investigating students’ learning behavior. Participation in the study was voluntary. All 59 students agreed to participate in the study and signed a written consent form, which also included the permission to utilize their data (made anonymous) in a scientific publication. Data were anonymized by having participants generate an individual code which they could regenerate in all of the experimental sessions. Students could opt out of having their data used for research at any time.

At the beginning of the semester, students were randomly assigned to the two groups. This resulted in 30 participating students in the open-book group and 29 in the closed-book group, 46 of which showed up for the surprise quiz, 27 from the open-book group (mean age = 20.96 years, 3 males plus 1 person who did not provide gender information) and 19 from the closed-book group (mean age = 20.53 years, 1 male). At the final module exam, which took place 8 weeks after the surprise quiz, 51 participants showed up (25 from the open-book group and 26 from the closed-book group)^1^. We did not ask for the personal code in the final module exam to maintain anonymity. Therefore, we could not relate the data collected in the module exam to the data collected in the surprise quiz.

### Materials and Procedure

#### General Course Information

The two seminars (“Cognition”) served to accompany the lecture “Introduction to cognitive psychology,” with which they formed the module “Cognitive psychology.” Right from the beginning of the lecture, students knew about the final exam at the end of the lecture. (Students were aware that this module exam would include both questions referring to matters exclusively taught in the lecture and matters taught in the seminars).

The students in both groups were told that they would receive practice questions addressing the content of the current seminar lesson. The two seminars were taught by the same instructor (AS, the third author) and the lessons followed the exact same predetermined script in both groups and used the same literature. The first and the last lessons of the experiment covered one psychological journal paper each, the remaining five lessons covered two papers each (see Table 1). In the first 5 min of each lesson, students could ask questions regarding the previous lesson (including regarding the practice questions). Then student presentations took place, which lasted 30 min for each paper and for which printouts of the slides were distributed to the students. The instructor closely supervised preparation of the presentations and of the handouts to ensure that the input in the two groups was of the same quality and informativeness. For the remainder of the lesson, there was time for discussion, except for the final 10 min, in which the practice tests took place.

#### Practice Tests

The critical manipulation concerned the final 10 min of seven lessons in each group. Here, students received between two and three short-answer questions on a sheet of paper that addressed central aspects of the papers that had been covered in the current lesson (16 questions in total). Students had to answer these questions by themselves with a few sentences. Both groups were given the exact same questions, but the open-book group was instructed to consult the provided learning materials and their own notes while answering these questions; the closed-book group was not allowed to use these materials. None of the participants received direct feedback regarding test performance but all of them kept the learning materials and were encouraged to restudy the materials at home. At the beginning of each lesson, students could ask questions regarding the previous lesson. These questions could also concern the practice test questions^2^.

#### Dependent Measures

One week after the last practice test, a *closed-book surprise quiz* took place, of which students were not informed beforehand and which was introduced as a practice quiz for the final module exam. Consequently, the delay between practice tests and surprise quiz ranged from 1 to 7 weeks. The surprise quiz included all 16 practice questions and was the same for both groups. Half of the 16 questions appeared in the same format; the other half was transformed into a different test format: one short-answer question was changed to four true-false statements each. After the surprise quiz, students received feedback regarding the correct answers. We expect that participants’ performance should differ with respect to the testing condition (closed book > open book).

In addition, we examined performance on the multiple choice questions of the official *final module exam,* which took place 8 weeks after the surprise quiz. The exam covered only the lecture, but three topics overlapped with the topics covered during the learning phase of the experiment. Specifically, the exam included 9 multiple choice items which referred to subject matter also covered during the 7 experimental lessons of the seminar and 21 multiple choice questions which referred to other subject matter (i.e., topics that were either part of the seminar lessons after the experimental lessons or that were solely covered in the lecture). Therefore, we analyzed performance on the exam questions that covered overlapping content (related exam questions) as an additional dependent variable to explore whether a potential retrieval practice effect transfers to related but different test questions. Performance for the nine questions addressing subject matter of the seven practice tests should differ with respect to the testing condition (closed book > open book). In an additional ANCOVA, we included performance on the remaining exam questions as a covariate to account for individual differences in general ability^3^. We will further report performance on the 21 unrelated exam questions.

## Results

### Surprise Quiz

#### Scoring

Maximally, participants could reach 32 points for short-answer questions (same format questions) and 32 points for true-false statements (changed format questions). The total score (in percent) served as the main dependent variable. The surprise quiz data were scored by a student assistant who was blind to the experimental condition.

#### Statistical analyses

Forty-six participants showed up for the surprise quiz, 27 from the open-book group and 19 from the closed-book group. We calculated the proportion of achieved points in the surprise quiz per group. The z-transformed raw values were submitted to a 2 × 2 mixed ANOVA with the between-subjects factor practice test type (closed-book vs. open-book) and the within-subject factor final test question type (short-answer vs. true/false). The ANOVA revealed a significant main effect for practice test type [*F*(1,44) = 5.15, *p* = 0.028, $\eta_{p}^{2}$ = 0.11] whereas the main effect for final test question type and the interaction did not reach significance (both *F*s < 1). Students who performed the practice tests in the closed-book condition performed significantly better (*M* = 45.09% correct answers, *SD* = 14.71) than those in the open-book condition (*M* = 36.73% correct answers, *SD* = 9.30).

### Module Exam

#### Scoring

Students could achieve either one point or zero points per question, which resulted in a maximum score of nine points for the questions analyzed here. The answers were scored by the first author of the manuscript (RR) who was blind to the experimental condition. Subsequently, the third author (AS) attributed these data (based on the students’ names) to one of the two experimental conditions.

#### Statistical analyses

Fifty-one students took part in the module exam, 25 from the open-book group and 26 from the closed-book group. Here, we analyzed the nine related multiple choice questions. The proportion of correct answers was higher for students in the closed-book group (*M* = 61.11%, *SD* = 13.05) than for students in the open-book group (*M* = 48%, *SD* = 17.19; *t(*49) = 3.08, *p* = 0.003, *d* = 0.86).

To control for differences in general ability, we also analyzed the effect of practice test type on performance for the related questions including performance on the unrelated questions as a covariate. Even with this covariate included, there was a significant advantage for the closed-book group over the open-book group, *F*(1,48) = 9.40, *p* = 0.004, $\eta_{p}^{2}$ = 0.16.

In addition, we analyzed performance on the 21 unrelated questions. Here, the two groups did not differ significantly (*M* = 58.44%, *SD* = 18.65 for open-book condition vs. *M* = 63.25%, *SD* = 15.72 for closed-book condition, *t*(49) = 1.03, *p* = 0.31), *d* = 0.28.

## Discussion

The present field experiment demonstrates a learning advantage of closed-book practice tests over open-book practice tests. As far as we know, this is the first time that a closed-book testing advantage over open-book testing was observed^4^. The closed-book effect observed in our study was not restricted to items which were practiced in the seven testing sessions and worked on again (in the same or slightly modified form) in the surprise quiz. Also in the module exam in which different, but related questions had to be answered, a closed-book advantage was found.

In the introduction of this paper, we stated that comparing learning performance of closed-book practice tests with open-book practice tests is the strongest test for the retrieval practice hypothesis. Thus, the finding that participants learning with a closed-book practice test outperformed those learning with an open-book practice test seems to support the theoretically highly relevant assumption that the testing effect is due to retrieval practice. This, however, raises the question why we found a retrieval practice effect but other researchers did not (Agarwal et al., 2008; Agarwal and Roediger, 2011).

First, a number of limitations of our field study need to be considered before interpreting our data. For one, the two seminars were taught by the third author of this paper, who was not blind to the experimental conditions while teaching. The lessons followed the exact same predetermined script in both groups and student presentations and printouts were strictly supervised to make the seminars as similar as possible. Nonetheless, slight differences in teaching cannot be excluded, which may have benefited the closed-book group for which an advantage had been predicted. However, the need to teach the two groups as similarly as possible also put high demands on the teacher. Therefore, we opted against trying to persuade an uninformed teacher who might have put less effort into following the script and supervising the student presentations. Nonetheless, it cannot be excluded that the predicted closed-book effect was (at least partly) due to demand characteristics.

A second limitation concerns the different numbers of participants in the two experimental groups in the surprise quiz. The fact that the proportion of students attending the surprise quiz was lower in the closed-book group than in the open-book group points to the possibility that the students in the two groups differed in some respect, for instance with respect to motivation. Such potential differences either might have existed beforehand or might even be a consequence of the experimental manipulation, if the students who had to perform the harder versions of the tests enjoyed the courses less. In this case, the higher scores for the closed-book group in the surprise quiz might also be due to selectivity of the sample because only the more motivated students in the closed-book group showed up for the session in which the surprise quiz took place. This is, however, not the case for the module exam, for which participation was obligatory and for which group sizes did not differ. What is more, the different attendance rates for the surprise quiz should have inverse effects on the exam data: As more students in the open-book group participated in the surprise quiz, this group should have benefited disproportionately high from the additional retrieval opportunity and the feedback in the surprise quiz. As the closed-book advantage showed up in the exam data as well, we assume that it cannot solely be attributed to the different attendance rates for the surprise quiz—at least when considering both dependent variables in combination.

A third objection is restricted to the analysis of the final module exam: The means for the related questions in the final exam (48 and 61%) were surprisingly low. Even though students had the opportunity to study for the exam and had taken practice tests along the way, performance was (numerically) worse for these questions than for the unrelated questions which had not been practiced in the seminars (58 and 63%). The related and unrelated questions were not matched with respect to difficulty and could not be matched with respect to the delay between coverage in class and the exam. As most of the critical sessions of the experiment took place in the first half of the semester, the delay between coverage of the topics and the final exam was shorter for the unrelated than for the related questions. This might have contributed to the overall difference between the question types. Nonetheless, the module exam data should be taken with a pinch of salt.

In addition to these limitations, a number of differences between our study and the studies by Agarwal et al. (2008) might be responsible for the different findings. One concerns the different delays between the practice tests and the final test. In case of Agarwal et al. (2008), the final test was presented 1 week after the learning session (which was also the case in Agarwal and Roediger’s, 2011, study). As suggested above, this final test delay might be too short to detect an effect of retrieval practice compared to a strong control condition (see also Rummer et al., 2017). Assuming that there is a reliable closed-book advantage in the present study despite its limitations, this can be interpreted as a retrieval practice effect because the practice tests only differed in the degree to which retrieval from long-term memory was required to answer the questions. Students could have benefited from retrieval practice either because memory traces were strengthened by elaborating the encoded information and by creating different retrieval routes to the information in long-term memory (e.g., Carpenter and DeLosh, 2006; Carpenter, 2009) or because practicing retrieval during learning constituted transfer-appropriate processing (e.g., Morris et al., 1977; Bjork, 1988; Roediger and Karpicke, 2006). Given that our study took place in a field setting and covered learning matter that was relevant for passing an exam, what happened in the practice tests could also have influenced students’ behavior both in and outside the classroom. In particular, we think that the two versions of the practice tests could have had *indirect* effects on learning performance *via* students’ motivation.

Even though students did not receive direct feedback after each practice test, performance in the closed-book tests should provide more diagnostic and more negative feedback than performance in the open-book tests in terms of noticing failures to retrieve the information from memory or less confidence in one’s answers. This difference may have led to indirect effects on students’ behavior. For one, it might be that the two groups differed with respect to the numbers of questions asked in the following lesson. At the beginning of each lesson, students had the opportunity to ask questions concerning the previous lesson, including the practice questions. Students in the closed-book group may have asked both more and more concrete questions than the students in the open-book condition because they had no opportunity to consult the materials when answering the questions. Unfortunately, we did not record the questions asked by the students so that we cannot test this (*post hoc*) assumption based on the current data set.

Another indirect effect of the closed-book test concerns the preparation and repetition of the learning matter at home. Since the content of the learning materials was highly relevant to the students in our study, it might well be that indirect feedback resulted in more extensive study at home in the closed-book group than in the open-book group. Therefore, it is plausible that the advantage for the closed-book condition over the open-book condition is (at least partly) due to longer and/or more intensive repetition of the learning matters at home. Unfortunately, we do not have any data to test this assumption empirically. This issue should be addressed in future studies either by having students write a learning diary or—preferably—by providing the learning materials online and logging the learning times.

These effects should be far less pronounced in laboratory studies investigating open-book versus closed-book practice tests, in which participants were presented with learning content that was not important to reach a goal of personal relevance for them (Agarwal et al., 2008; Agarwal and Roediger, 2011; Roelle and Berthold, 2017). Consequently, potentially more negative feedback in the closed-book groups would not have led to extensive study at home in these experiments. Different degrees of motivational effects might therefore explain the difference between our findings and earlier laboratory studies. The results of Gharib et al. (2012) field experiment in which open-book and closed-book tests in a university course did not affect performance in a later test might speak against this interpretation. However, in their study, the “practice tests” were actual exams and the test that served as the dependent variable was a delayed (surprise) retention quiz. In this setting, it is not very probable that the students relearned the materials following the course exam, passing which was their main purpose. Thus, the test format of the course exam should not have influenced students’ behavior between the manipulation and the final test.

Based on these considerations, it is not justified to interpret our findings solely in terms of a direct retrieval practice effect. Nonetheless, our results suggest that open-book and closed-book testing are not equivalent, at least from an applied point of view. But is it justified to give a strict recommendation to use closed-book tests instead of open-book tests based on our experiment? Given the small number of participants, the restriction to two seminars in cognitive psychology, and the limitations outlined above, we are cautious here. What is needed is more research comparing open-book and closed-book testing in field experiments using real learning materials. These field experiments should address various kinds of learners (at school and at university) as well as various kinds of subjects. In addition, these experiments should track the time learners spend at home with the provided learning materials.

## Ethics Statement

This study was carried out in accordance with the recommendation of the ethics committee of the Faculty of Educational Sciences of the University of Erfurt with written informed consent from the subjects in accordance with the Declaration of Helsinki. In Germany, there is no general obligation to get an approval of the local ethics commitee for psychological studies (at least as long as they are not invasive). Yet, the study reported here was conducted in line with the ethical standards of the American Psychological Association (APA) and the German Association of Psychology (DGPs), which are in accordance with the Declaration of Helsinki.

## Author Contributions

RR and JS wrote the manuscript and contributed to theoretical conceptualization. RR, JS, and AS analyzed the data. RR and AS planned the field experiment. RR adviced during the seminars. AS held the classes.

### Conflict of Interest Statement

The authors declare that the research was conducted in the absence of any commercial or financial relationships that could be construed as a potential conflict of interest.

## Footnotes

^1^A smaller number of students participated in the surprise test than in the final module exam. This is probably the case because participation in the seminar lessons (including the one with the surprise test) was voluntary, while taking the exam was obligatory. In addition, eight of the 59 students missed the exam due to illness. As it was not possible to present the same questions at the second exam date, only the data for those students who attended the first exam were included in our study.

^2^Unfortunately, the questions asked by the students were not recorded.

^3^As stated above, the final module exam data could not be related to the surprise quiz data because participants did not provide their codes for the exam (which would have compromised anonymity).

^4^A partial exception is the study by Roelle and Berthold (2017), who found a closed-book advantage for questions of low complexity but an open-book advantage for complex questions.
